# Probabilistic Mapping of Magnetic Resonance‐Guided Focused Ultrasound (MRgFUS) Thalamotomy Targets in Essential Tremor and Tremor‐Dominant Parkinson's Disease: Insights from a German Cohort

**DOI:** 10.1002/mds.70003

**Published:** 2025-08-08

**Authors:** Jonas Krauss, Neeraj Upadhyay, Veronika Purrer, Valeri Borger, Marcel Daamen, Hannah Weiland, Angelika Maurer, Carsten Schmeel, Alexander Radbruch, Markus Essler, Ullrich Wüllner, Henning Boecker

**Affiliations:** ^1^ Clinical Functional Imaging Group, Department of Nuclear Medicine University Hospital Bonn Bonn Germany; ^2^ Department of Parkinson, Sleep and Movement Disorders University Hospital Bonn Bonn Germany; ^3^ German Center for Neurodegenerative Diseases (DZNE) Bonn Bonn Germany; ^4^ Department of Neurosurgery University Hospital Bonn Bonn Germany; ^5^ Department of Neuroradiology University Hospital Bonn Bonn Germany

## Abstract

Precise targeting in magnetic resonance‐guided focused ultrasound (MRgFUS) is critical for effective tremor control in essential tremor and tremor‐dominant Parkinson's disease, as small deviations can reduce efficacy or cause side effects. In our cohort, sweetspots were identified at Montreal Neurological Institute (MNI) coordinates *x* = −12.4, *y* = −17.5, *z* = −1.7 (ET) and *x* = −13.4, *y* = −19.8, *z* = −3.0 (TDPD).

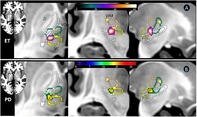

Magnetic resonance‐guided focused ultrasound (MRgFUS) thalamotomy is a non‐invasive alternative to deep brain stimulation (DBS) for the treatment of essential tremor (ET) and tremor‐dominant Parkinson's disease (TDPD). While DBS outcomes have improved through systematic mapping of lead placement, similar precision in lesion targeting may be crucial for optimizing MRgFUS efficacy^1^, ^2^ as even small deviations from optimal spots may result in reduced efficacy, relapse, or side effects.^3^, ^4^ We present new findings from a German cohort (54 ET,^5^ 27 TDPD^6^ patients) treated at University Hospital Bonn between 2019 and 2025, applying probabilistic sweetspot mapping.

To explore lesion efficacy, we performed voxel‐wise mapping of percentage tremor improvement on the treated side 6 months post‐treatment (ET: Fahn–Tolosa–Marin Clinical Rating Scale for Tremor [FTM]‐A/B; TDPD: Unified Parkinson's Disease Rating Scale‐Part III [UPDRS‐III] tremor subscore, items 15–18), following methods from previous literature.^1^ Statistical significance was assessed with Wilcoxon signed‐rank tests (*P* < 0.05), and permutation testing (n = 1000) was applied to validate the robustness of the results.

We observed high clinical response rates, with a mean tremor improvement of 68.8% in ET and 37.0% in TDPD at 6 months post‐treatment. To holistically evaluate treatment effects, we generated voxel‐wise odds ratio (VOR) maps^7^ for common adverse effects, including paresthesia, gait disturbance, taste disturbance, and dyskinesia.

In ET, the tremor sweetspot was localized at Montreal Neurological Institute (MNI) coordinate *x* = −12.4, *y* = −17.5, *z* = −1.7, while in TDPD, it was identified at *x* = −13.4, *y* = −19.8, *z* = −3.0. FTM‐C sweetspots for functional abilities were located at *x* = −15, *y* = −18.2, *z* = −2.8 in ET and *x* = −13.6, *y* = −19.8, *z* = −3 in TDPD, with substantial overlap with their respective tremor sweetspots. In ET, the tremor sweetspot notably overlapped with side effect maps for paresthesia and dyskinesia, suggesting these complications may reflect inherent risks of lesioning this region. Dyskinesias were associated with more dorsolateral lesion locations (*x* < −13.5, *z* > −1.5), implicating the zona incerta and ventroposteromedial nucleus (VPM) border as a critical region. Conversely, gait disturbances (in ET) and taste disturbances (in both ET and TDPD) appeared more likely with posterior and superior lesions, and may be avoidable when targeting more ventral (−3 < *z* < −1) and anterior (*y* < −18) regions.

By subtracting side effect maps from the tremor sweetspot in ET, we identified a lateral‐anterior region within the ET sweetspots showing a high efficacy‐to‐risk ratio. This area also aligned with the FTM‐C sweetspot in ET, while our TDPD tremor sweetspot was located more ventral and posterior. The overall convergence suggests a shared effective lesioning zone across both conditions (Fig. 1). While the ET sweetspot aligns with prior literature,^2^ our TDPD sweetspot—although effective—was situated more ventrally and posteriorly than previously reported optimal coordinates,^1^ which were associated with greater tremor reduction, suggesting that more anterior (*x* = 15) and dorsal (*z* = 1.5) targeting may be preferable in TDPD.

**FIG. 1:**
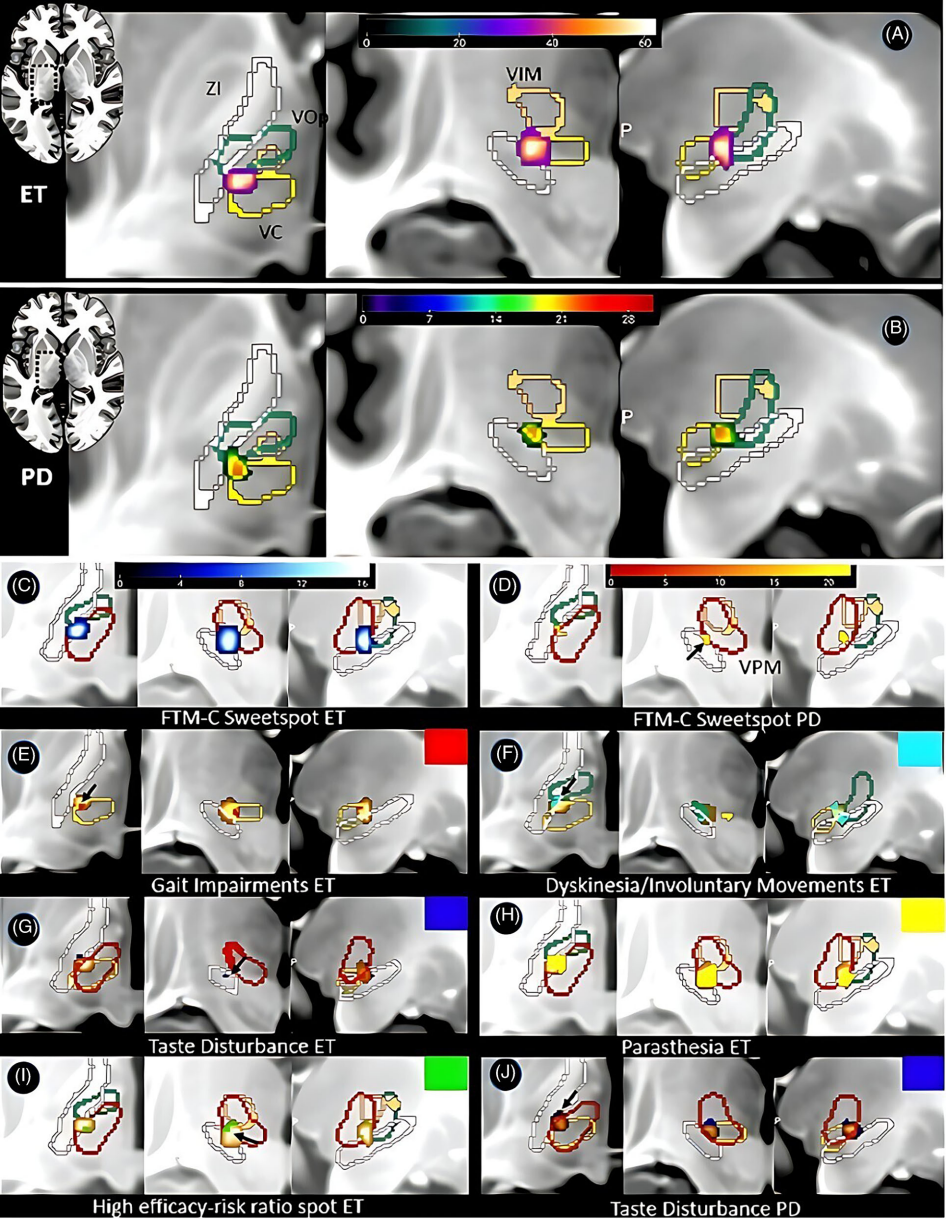
Probabilistic mapping of clinical improvement scores and side effect voxel‐wise odds ratios (VORs). Sweetspots for tremor and functional improvement in (A + C) essential tremor (ET) (−12.4/−17.5/−1.7) and (B + D) Parkinson's disease (PD) (−13.4/−19.8/−3.0). Maps (E–J) show side effect VORs; (I) displays ET zones with good effect–risk ratio after subtracting VOR maps from sweetspot map. Key: yellow, nucleus ventro‐caudalis (VC); green, nucleus ventro orealis posterior (VOp); white, zona incerta (ZI); copper, nucleus ventralis intermedius (VIM); red, ventral posterior‐medial nucleus (VPM). FTM, Fahn–Tolosa–Marin Clinical Rating Scale. [Color figure can be viewed at wileyonlinelibrary.com]

While these findings may help refine the efficacy–risk balance in thalamic MRgFUS targeting, they should not be interpreted as strict anatomical boundaries. Multicenter validation remains warranted.

## Author Roles

(1) Research Project: A. Conceptualization, B. Design, C. Organization, D. Execution, E. Review and Critique; (2) Statistical Analysis: A. Design, B. Execution, C. Review and Critique; (3) Manuscript Preparation: A. Writing of the First Draft, B. Review and Critique.

J.K.: 1A, 1B, 1C, 2A, 2B.

N.U.: 1A, 2B.

V.P.: 1C, 2B.

V.B.: 1C, 2B.

M.D.: 1B, 2B.

H.W.: 1C, 2B.

A.M.: 2B.

C.S.: 2B.

A.R.: 2B.

M.E.: 2B.

U.W.: 1C, 2B.

H.B.: 1A, 1B, 2B.

All authors provided their final approval and are accountable for all aspects of the work in ensuring that questions related to the accuracy or integrity of any part of the work are appropriately investigated and resolved.

## Data Availability

The data that support the findings of this study are available from the corresponding author upon reasonable request.
